# Carrion’s disease: more than a neglected disease

**DOI:** 10.1186/s13071-019-3390-2

**Published:** 2019-03-26

**Authors:** Meritxell Garcia-Quintanilla, Alexander A. Dichter, Humberto Guerra, Volkhard A. J. Kempf

**Affiliations:** 1 University Hospital, Goethe-University, Institute for Medical Microbiology and Infection Control, Frankfurt am Main, Germany; 2 Universidad Peruana Cayetano Heredia and the Instituto de Medicina Tropical Alexander von Humboldt, Lima, Peru

**Keywords:** *Bartonella bacilliformis*, Carrion’s disease, Vector-borne disease, *Lutzomyia*, South America, Neglected tropical disease

## Abstract

Infections with *Bartonella bacilliformis* result in Carrion’s disease in humans. In the first phase of infection, the pathogen causes a hemolytic fever (“Oroya fever”) with case-fatality rates as high as ~90% in untreated patients, followed by a chronical phase resulting in angiogenic skin lesions (“verruga peruana”). *Bartonella bacilliformis* is endemic to South American Andean valleys and is transmitted *via* sand flies (*Lutzomyia* spp.). Humans are the only known reservoir for this old disease and therefore no animal infection model is available. In the present review, we provide the current knowledge on *B. bacilliformis* and its pathogenicity factors, vectors, possible unknown reservoirs, established and potential infection models and immunological aspects of the disease.

## Background

Carrion’s disease is a vector-borne biphasic illness restricted to the South American Andes including Peru, Ecuador and Colombia and is endemic in Andean valleys at an altitude of 600–3200 m above sea level; it has also been described in the coastal areas of Guayas and Manabi in Ecuador [1, 2]. The causative agent of this neglected disease is *Bartonella bacilliformis*, which is a motile, aerobic, facultative intracellular alpha-2-proteobacterium. It infects human erythrocytes first causing a serious acute hemolytic anemia called “Oroya fever” followed by a chronic infection of endothelial cells resulting in vasculo-endothelial proliferations called “verruga peruana” as the result of the continuous angiogenic stimulus by *B. bacilliformis*. These two syndromes typically occur sequentially but sometimes independently. An infection with *B. bacilliformis* can result in a variety of different clinical manifestations such as severe illness, mild or asymptomatic illness or chronic asymptomatic bacteremia [3]. The exact factors which define the clinical course of Carrion’s disease are still unknown but it is assumed that the interplay of virulence factors of the strain, the inoculum and the fitness and individual predisposition of the host determine the severity of the clinical manifestation [4]. The existence of less virulent bacterial strains that cause mild atypical bartonellosis has been suggested, meaning Carrion’s disease is under-reported [1]. *Bartonella bacilliformis* is transmitted to humans by female phlebotomine sand flies (*Lutzomyia* spp.) which are present in high-altitude regions. Climatic changes favor the expansion of *B. bacilliformis* infections through sand fly proliferation [5, 6].

**Fig. 1:**
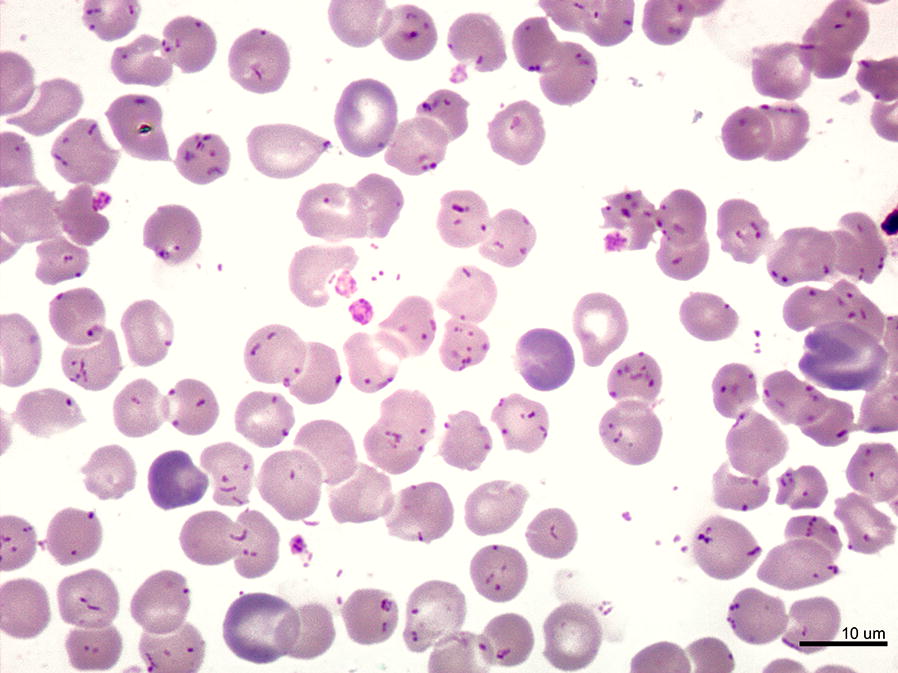
Overwhelming parasitism of erythrocytes by *B. bacilliformis*. Giemsa-stained blood smear from a patient with Oroya fever, showing parasitism of all erythrocytes, with bacillary and coccoid forms of *B. bacilliformis. Scale-bar*: 10 µm (courtesy of P. Ventosilla and M. Montes, Universidad Peruana Cayetano Heredia, Lima, Peru)

Oroya fever (characterized by an intraerythrocytic anemia) (Fig. 1) is more common in children than in adults and it is characterized by a plethora of symptoms including fever, hemolytic anemia, pallor, myalgia, headache, anorexia, tachycardia and hepatomegaly [5] with an immune-compromised state that facilitates secondary infections such as *Toxoplasma gondii* myocarditis or bacteremia with *Staphylococcus aureus* or *Salmonella enterica* [4]. In this early phase of infection, *B. bacilliformis* spreads into the circulatory system invading erythrocytes and leading a hemolytic anemia due to the splenic depletion of infected erythrocytes. Case-fatality rates as high as 88% have been described in the Oroya fever phase in untreated patients, meanwhile around 10% case-fatality rates have been reported for patients receiving timely antibiotic treatment [7].

The life-cycles of *Bartonella* spp. in their respective vectors are better known for many of the species other than *B. bacilliformis*. Those studies propose that *Bartonella* is present in the midgut of arthropod vectors and is released onto the mammalian skin in feces in order to pass to the dermal niche after erosion of the skin. The lymphatic system seems to be responsible for spreading the pathogen into the circulatory system and an intracellular presence of the bacteria (here in erythrocytes) avoids clearance by the host immune system [8, 9]. In the case of *B. bacilliformis*, it remains unknown if there is a dermal inoculation prior the blood spreading since the only known vectors to date are sand flies (*Lutzomyia* spp.) which might transmit the bacteria directly into the bloodstream. Moreover, as there are currently no animal infection models, the exact mechanisms underlying the pathobiology of this early infection state cannot be analyzed in detail in an experimental setting.

**Fig. 2:**
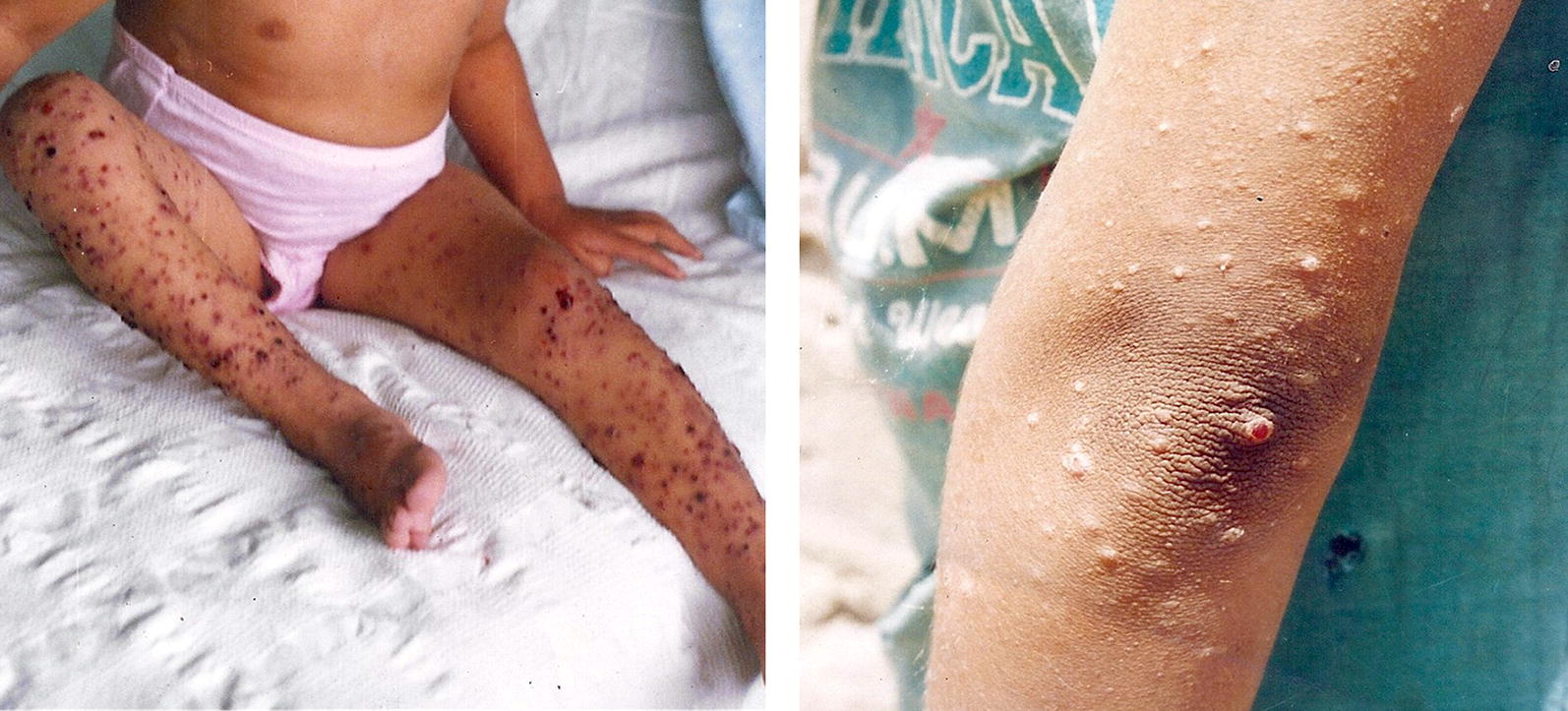
Patients with Verruga peruana caused by *B. bacilliformis*. Left: 9-year-old girl with numerous bleeding verrugas on her legs; Huaraz, Ancash, 1993. Right: 17-year-old girl (facing left) showing multiple verrugas close to her left elbow; a single verruga has broken the overlying epidermis, and may later bleed; Huari, Ancash, 2002 (courtesy of C. Maguiña, Universidad Peruana Cayetano Heredia, Lima, Peru)

**Fig. 3:**
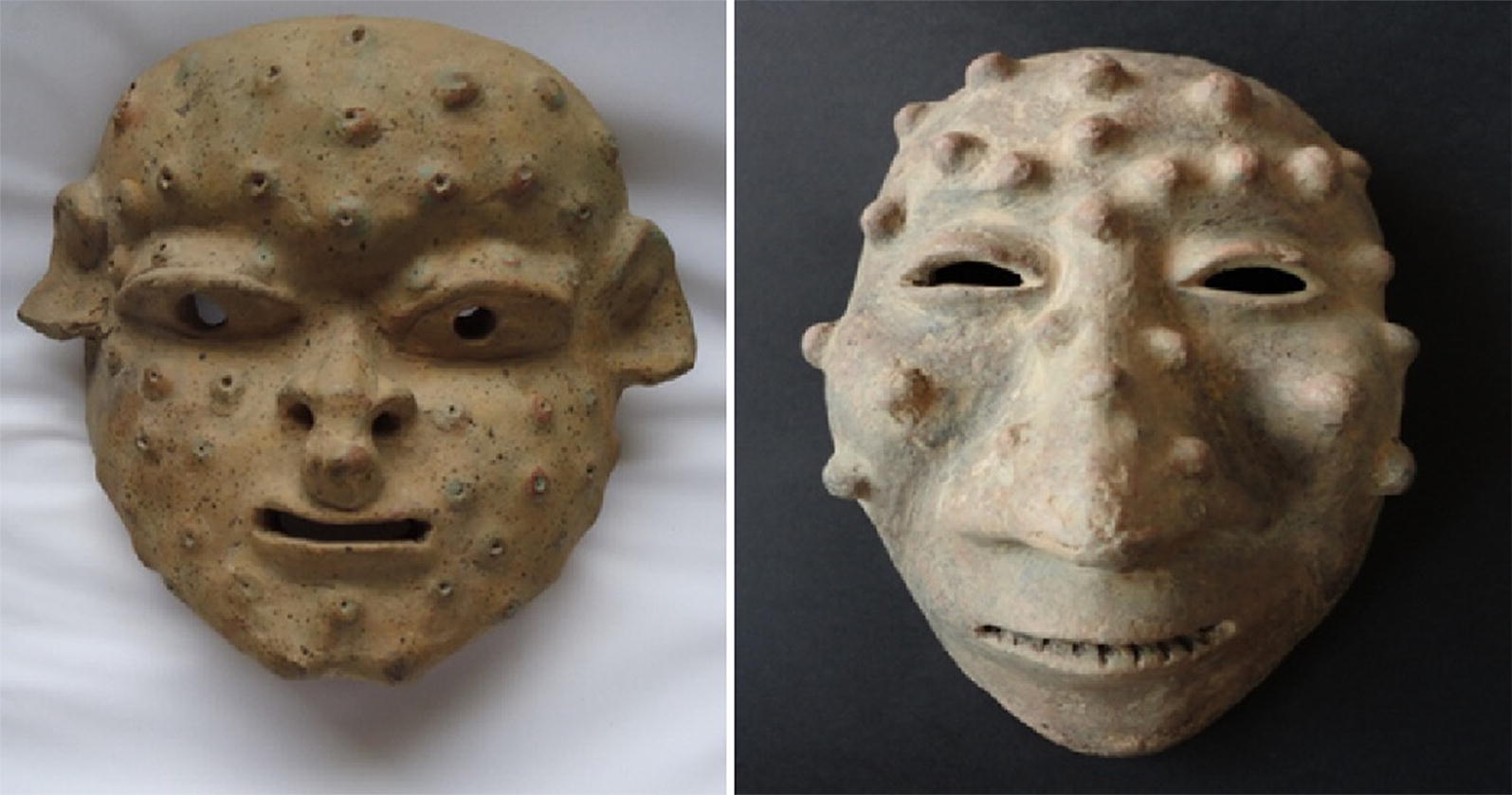
Ceramic masks (400 B.C.–400 A.D.). Two masks discovered in Ecuador displaying the facial symptoms of verruga peruana. Citation: Sotomayor-Tribín HA. Pensamiento analógico mítico en la interpretación del arte prehispánico de interés para la arqueomedicina y la paleopatología. Repert Med Cir. 2016;25:50–71 [94]. With permission of Elsevier

If Oroya fever is survived, the chronic verruga peruana phase can occur impressing as blood-filled nodular hemangioma-like lesions in the skin (Fig. 2). Under all human pathogenic bacteria, only the family of *Bartonella* has the ability to trigger angiogenic disease entities (*B. bacilliformis*: verruga peruana; *B. henselae*, *B. quintana*: bacillary angiomatosis, peliosis hepatis [10]). It is suggested that the abnormal endothelial cell proliferation is induced by a chronic *Bartonella*-infection in which the bacteria are included into vacuoles inside the capillary endothelium. Peruvian warts are mostly found on the head and extremities persisting from weeks to months. These lesions were described in the 16th century by Spanish conquerors [5, 7] (Fig. 3).

In general, Carrion’s disease has been only poorly investigated; a PubMed query in December 2018 with the terminus “*Bartonella bacilliformis*” revealed only 258 publications, many of them from Peru where the pathogen is endemic [in contrast: *Staphylococcus aureus*, 112,157 publications; *Trypanosoma cruzi* (endemic in South America), 14,936 publications). The field suffers from a significant lack of data about many aspects of Carrion’s disease, a limited knowledge about confirmed vectors or reservoirs of *B. bacilliformis* and the absence of feasible animal infection models. The assumed general strategy underlying a *Bartonella* infection is (i) the avoidance of the host immune response and the infection of a primary niche (if this exists); (ii) the invasion of erythrocytes; and (iii) an intraerythrocytic replication [11] resulting in erythrocyte rupture [12]. Exact mechanisms involved in all these steps are not studied in detail. It is known that flagella of *B. bacilliformis* are not recognized by Toll-like receptor 5 (TLR5) avoiding a broad activation of the innate immune system [13] and it is assumed that adhesins might mediate autoaggregation [14] to prevent phagocytosis [11]. On the other hand, adhesins, flagellin, hemolysin, deformin or the invasion associate locus proteins A and B are some factors that have been associated with erythrocyte infections. In this review we summarize the current knowledge for *B. bacilliformis* with regard to vectors, pathogenicity factors and infection models.

## Vectors and reservoirs for *B. bacilliformis*

**Fig. 4:**
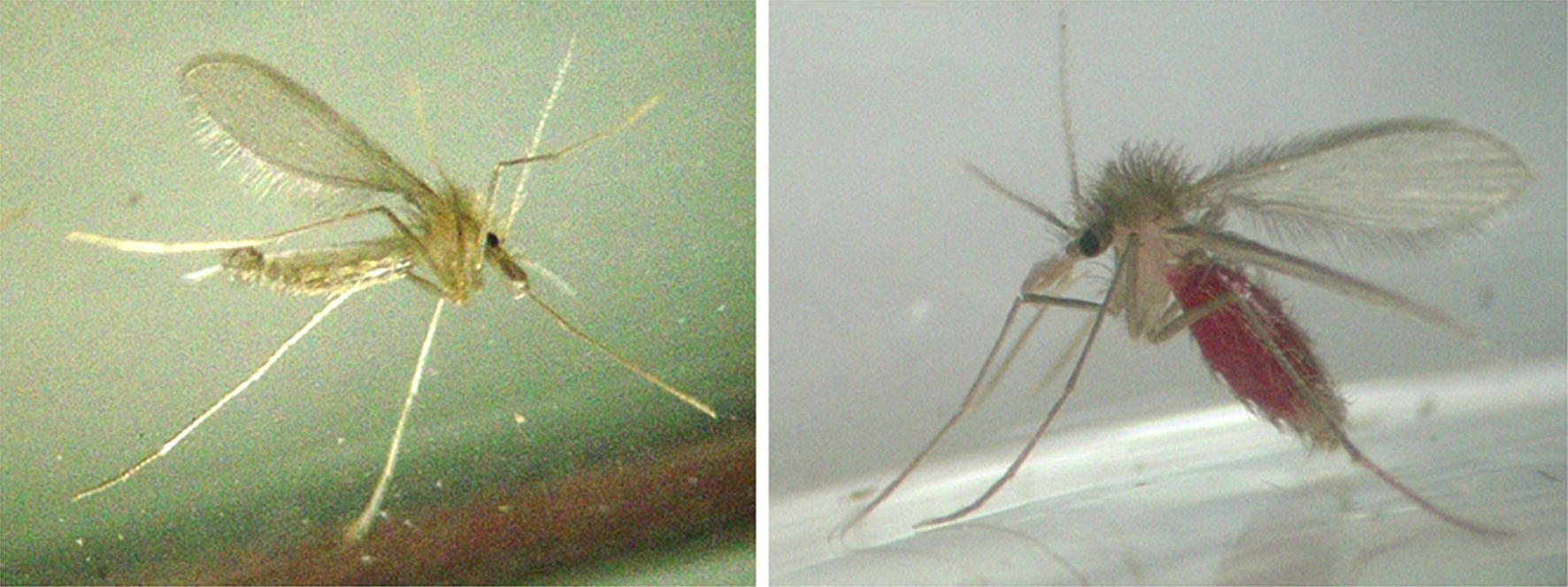
Adult *Lutzomyia verrucarum* sand flies. Left: male. Right: blood-fed female. Colony-bred adults. Length of each between 2 and 3 mm (courtesy of E. Pérez, Universidad Peruana Cayetano Heredia, Lima, Peru)

Sand flies belonging to the genus *Lutzomyia* (Fig. 4) are considered the only vector for *B. bacilliformis*. The first evidence for the transmission of *B. bacilliformis* was found in 1913 when Charles Townsend captured sand flies in the train station where workers suffered from Carrion’s disease [15]. In 1929, the pioneer in analyzing Oroya fever, Hideyo Noguchi, determined which insects are responsible of the transmission of the disease by exposing *Macacus rhesus* monkeys to bat flies, bedbugs, buffalo gnats, fleas, horse flies, lice, mites, midges, mosquitoes, sheep ticks, ticks, and three species of sand flies (*L. verrucarum*, *L. peruensis* and *L. noguchii*). He injected crushed arthropods intradermally and blood cultures were analyzed for the presence of *B. bacilliformis.* The only vectors whose injections resulted in an infection were *L. verrucarum* and *L. noguchii* [16]. From literature, the following *Lutzomyia* species are suggested vectors for *B. bacilliformis*: *L. ayacuchensis* [2], *L. columbiana* [17], *L. gomezi* [17], *L. maranonensis* [18], *L. noguchii* [16], *L. panamensis* [17], *L. peruensis* [19, 20], *L. pescei* [5], *L. robusta* [21], *L. serrana* [2] and *L. verrucarum* [22]. However, the presence of *B. bacilliformis* DNA in these insects has only been demonstrated for *L. verrucarum* [22], *L. peruensis* [20], *L. robusta* [23] and *L. maranonensis* [18].

Colonization experiments with artificially-infected *L. verrucarum* (competent vector) and *L. longipalpis* (non-competent vector) showed that green-fluorescent protein (GFP)-expressing *B. bacilliformis* bacteria remain in the midgut and are digested with time in *L. longipalpis* (non-competent vector) meanwhile the pathogen is able to persist in *L. verrucarum* [24]. The molecular mechanisms for persistence in *L. verrucarum* have not yet been elucidated.

There is a clear correlation between the distribution of Carrion’s disease and the presence of vectors in endemic areas. The main sand fly species in northern, southern and central Peru are *L. verrucarum* and *L. peruensis*. These sand fly species are predominant at altitudes between 1100 and 3200 m above sea level in the Andean mountain valleys of South America [25, 26]. The epidemiological presence of Carrion’s disease in other areas, however, suggests the existence of other *Lutzomyia* vectors. *Lutzomyia serrana* was detected in an outbreak in Monzon Valley, *L. robusta* in outbreaks taking place in Jaen, San Ignacio and Utcubamba, and *L. pescei* in Huancavelica, Churcampa, Tayacaja, Urubamba, Calca and Quispicanchis (all Peru) during outbreaks [27]. In Colombia, the potential vector for Carrion’s disease is *L. columbiana*. During 2009–2013, a total of 1389 cases of bartonellosis were reported in Colombia from which 16% were assigned to Carrion’s disease (~3% Oroya fever and ~13% verruga peruana). Reports demonstrated that it was not only the typical endemic areas such as Nariño, Cauca and Valle del Cauca that were affected, but also Antioquia, Caldas, Huila, La Guajira and Risaralda which were not previously considered to be endemic [28].

Noguchi suggested already in 1926 that ticks might represent possible vectors for *B. bacilliformis* as he demonstrated that *B. bacilliformis* was transmitted by bites of *Dermacentor andersoni* from two experimentally-infected to two healthy *Macacus rhesus* monkeys [29]. In a recent study, *B. bacilliformis* DNA was detected in ticks (*Amblyomma* spp. and *Rhipicephalus microplus*) collected from *Tapirus terrestris* and *Pecari tajacu* from Madre de Dios (Peru) suggesting that ticks might be at least considered as potential vectors for *B. bacilliformis* [30]. It is important to critically discuss some points of this study the possibility of false positive results due to the DNA extraction method (from crushed insects) or due to the high number of cycles (*n* = 55) and the missing amplicon sequencing procedures. A recent study identified a novel “*Candidatus* Bartonella rondoniensis” from kissing bugs (*Eratyrus mucronatus*) in French Guiana [31]. This novel strain is phylogenetically related to *B. bacilliformis* and *B. ancashensis*, both known to be human pathogenic [32]. More studies are needed to clarify whether *B. bacilliformis* and closely related species can be transmitted through other vectors to humans which are not assigned today.

Currently, apart from humans, there is no confirmed reservoir for *B. bacilliformis*. No solid evidence exists that *Tapirus terrestris* and *Pecari tajacu* might serve as reservoirs for *B. bacilliformis* because no serum/blood was collected from these two wild mammals from which *B. bacilliformis* DNA-positive ticks were removed [30]. On the other hand, the broad distribution of *Tapirus terrestris*, *Pecari tajacu* and ticks is not in concordance with the distribution of Carrion’s disease; therefore, further studies are needed to confirm or discard this possibility. In the hypothetical case that these wild animals did not suffer from a *B. bacilliformis* infection, ticks might have become infested *via* blood meals from other, so far unknown animals or even from humans since only 3 out of 43 ticks (6.97%) collected from three *Tapirus terrestris* and 12 out of 67 ticks (17.91%) collected from three *Pecari tajacu* were positive for *B. bacilliformis* DNA [30]. In the case that an animal is found to be bacteremic with *Bartonella* spp., one could assume that the majority of these blood-sucking ticks would harbor *B. bacilliformis* DNA as this has been demonstrated for feeding *Ixodes ricinus* ticks collected from a *B. henselae*-seropositive cat [33].

Many *Bartonella* species have various specific animal reservoirs (e.g. cats, deer, foxes, rodents, cattle [34]). For *B. bacilliformis*, some animal and plant reservoir candidates have been proposed in the past. Here, it is important to know that both male and female sand flies feed on plants, but only females feed on blood since blood meals are required for the maturation of eggs. [9]. A total of 50 animals were tested from households whose children were suffering from Carrion’s disease and only four out of nine non-domesticated rodents were found to be positive for *Bartonella*-like bacteria; unfortunately, no species determination was undertaken, so it remains unknown if an unexplored animal reservoir for *B. bacilliformis* might exist [35]. On the other hand, several human pathogens are able to infect or to persist on plant reservoirs such as *Salmonella enterica*, *Pseudomonas aeruginosa*, *Burkholderia cepacia*, *Erwinia* spp., *Staphylococcus aureus*, *Escherichia coli* and *Listeria monocytogenes* [36]. With this scenario, another possibility might be that *B. bacilliformis* survives in a plant environment and sand flies become infested after feeding from plants. Bacterial type III and type IV secretion systems are usually involved in plant infection processes. However, *B. bacilliformis* lacks these secretion systems [37]. In 1953, Herrer [38] tried to recover *B. bacilliformis* from euphorb plants distributed in the same areas where there had been recent cases of Carrionʼs disease where Carrion’s disease took place but without success.

## Pathogenicity factors of *B. bacilliformis*

The genus *Bartonella* can be classified into three clades which are formed by *Bartonella apis*, *Bartonella tamiae* and the eubartonellea. [39]. The most ancestral *Bartonella* spp., *B. apis*, is a honey bee gut symbiont. It is the only non-pathogenic representative of the genus *Bartonella* and the closest known relative of pathogenic *Bartonella* species. The genome of the intraerythrocytic pathogen *B. tamiae* shows many ancestral characteristics but lacks the most of the eubartonellea specific virulence factors. It is believed that this species presents the evolutionary transition state from a gut symbiont towards an intraerythrocytic pathogen [39]. The clade of the eubartonellea itself is subdivided in four major lineages (L1-L4). L1 is formed by *B. bacilliformis* and *B. ancashensis* and it is supposed that these *Bartonella* spp. infect exclusively humans. L2 species are restricted to ruminants and L3 and L4 species infect a variety of different reservoir hosts with the most commonly recognized human pathogenic species *B. henselae* and *B. quintana* (both members of L4). All members of the clade eubartonella harbour type IV secretion systems (T4SS) (VirB/VirD4, Vbh/TraG and/or Trw) for, e.g. cellular invasion. The only exception is *B. bacilliformis* which is the most ancestral species of this clade identified from phylogenetic studies. Genome evolution in *Bartonella* at species level shows that a high dynamic genomic expansion exists in some species (e.g. *B. tribocorum*: 2.64 Mb) and genome reduction in others as (e.g. *B. bacilliformis*: 1.45 Mb) [40].

## Confirmed pathogenicity factors of *B. bacilliformis*

### Adhesin

**Fig. 5:**
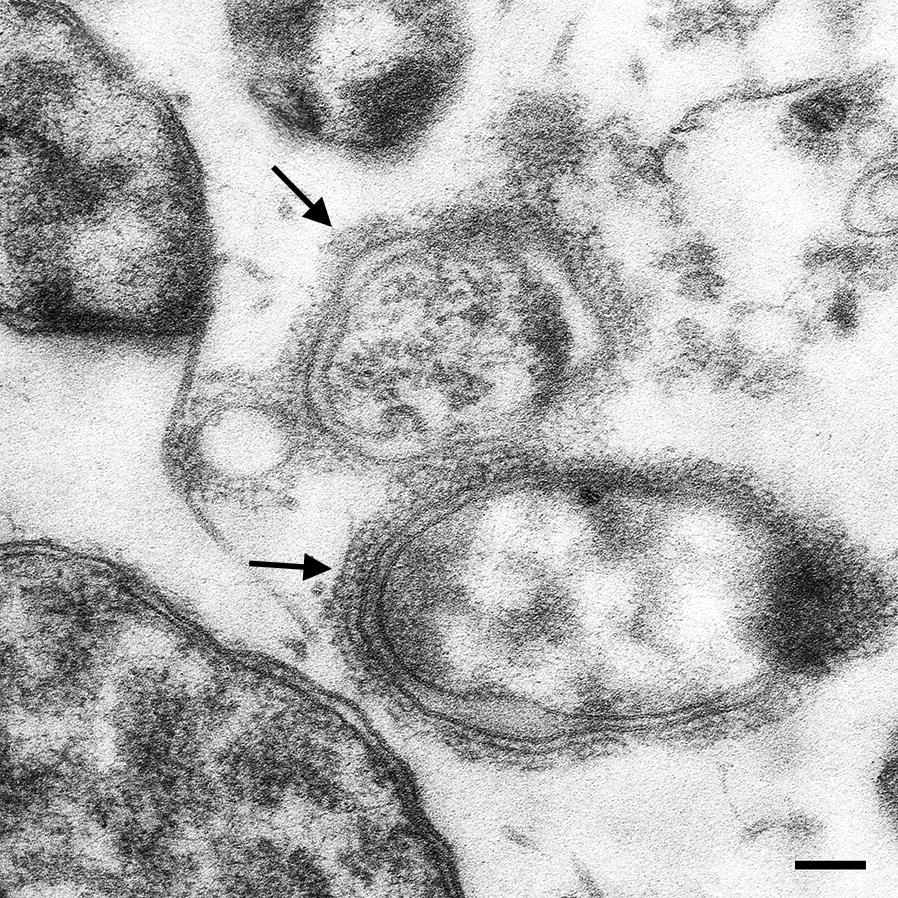
*Bartonella bacilliformis* adhesin A (BbadA) expressed on the surface of *B. bacilliformis*. Electron microscopy of *B. bacilliformis* ATCC 35686 (grown for four days at 28 °C in *Bartonella* liquid medium [95]). Arrows indicate the presumptive BbadA expression on the bacterial surface. *Scale-bar*: 100 nm (courtesy of M. Schaller and B. Fehrenbacher, Eberhard Karls-University, Tuebingen, Germany)

Trimeric autotransporter adhesins (TAA) are found in many Gram-negative bacteria. TAAs mediate autoaggregation, adherence to host cells and matrix proteins, are immunodominant and involved in triggering a specific host cell response after infection [14]. The essential role of TAAs in bacterial pathogenicity has been shown for several TAAs, such as *Yersinia* adhesin A (YadA) from *Y. enterocolitica* [41] or *Neisseria* adhesin A (NadA) from *N. meningitidis* [42]. As known today, TAAs are encoded in the genomes of all *Bartonella* spp. [10] and the best studied TAA is *Bartonella* adhesin A (BadA) of *B. henselae* [43–45]. Genes homologous to *badA* have also been found in the genomes of *B. bacilliformis* [10]. Here, three putative *B. bacilliformis* adhesins were identified (NCBI accession numbers WP_005766217.1, WP_005766221.1, WP_005767360.1) with a deduced TAA domain structure similar to other TAAs from species of the genus *Bartonella*. The exact role of *Bartonella bacilliformis* adhesin A (BbadA) in the infection process is not clear, own ongoing work is aimed to elucidate this in detail (Fig. 5).

### Flagellin

**Fig. 6:**
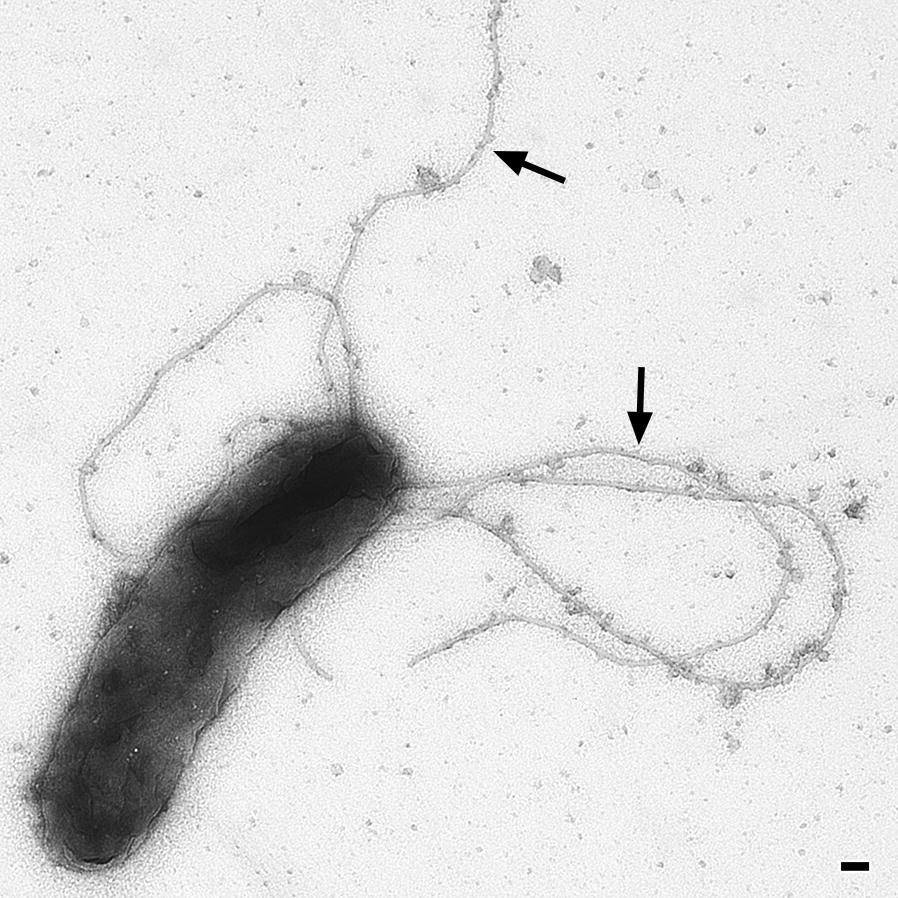
Flagella of *B. bacilliformis.* Electron microscopy of *B. bacilliformis* ATCC 35686 (grown for four days at 28 °C in *Bartonella* liquid medium [95]). Arrows indicate the presumptive BbadA expression on the bacterial surface. *Scale-bar*: 100 nm (courtesy of M. Schaller and B. Fehrenbacher, Eberhard Karls-University, Tuebingen, Germany)

Flagella mediate the motility of *B. bacilliformis* and are composed of 42 kDa flagellin subunits (NCBI accession number WP_011807398) [3]. Typically, *B. bacilliformis* expresses 2–16 unipolar flagella [3] ~3–10 µm in length (Fig. 6). Adherence of bacteria to erythrocytes correlates with their ability to be motile; however, it is not known whether flagella are directly involved in erythrocyte adhesion or if the bacterial motility increases the probability of encountering erythrocytes. Mutants lacking flagellin expression have been demonstrated to exhibit less erythrocyte adherence compared with wild type bacteria [46] and were unable to enter erythrocytes [47]. In accordance, it was reported that expression of flagella is decisive for erythrocyte invasion since the presence of anti-flagellin antibodies reduced *in vitro* the erythrocyte invasion of *B. bacilliformis* [48]. In contrast to other flagellated bacteria (e.g. *E. coli*, *P. aeruginosa* or *Legionella pneumophila*), flagellin from *B. bacilliformis* is not recognized by Toll-like receptor 5 (TLR5) due to an amino acid exchange in the N-terminal D1 domain and this avoids a NF-κB-regulated inflammatory host cell activation [13].

### GroEL

GroEL is a housekeeping protein found nearly in all prokaryotic cells. This heat-shock chaperone is highly conserved and its encoding sequence has been used for multi-locus sequence typing (MLST) [49] and for the analysis of phylogenetic relationships in *Bartonella* species [50]. The protein (NCBI accession number WP_005767840.1) is also immunodominant in humans [51]. GroEL is present in the inner and outer membrane of *B. bacilliformis* but it has also been reported to be secreted and involved in establishing an angiogenic phenotype of endothelial cells *in vitro* [52]. It remains unknown if GroEL is a mitogenic factor by itself or whether it interferes with the expression or stability of other angiogenic *B. bacilliformis* proteins. Secretion of GroEL has also been described in *Helicobacter pylori* to protect secreted ureases [53, 54]. The *groESL* operon is upregulated in response to thermal stress resulting in a ~4-fold induction of *groEL* expression by a temperature upshift from 30 °C to 37 °C comparable to the temperature shift occurring at the transmission event from sand fly vectors to the human host [55]. GroEL of *B. bacilliformis* increases apoptosis of human umbilical vein endothelial cells (HUVEC) [56] thereby possibly regulating the growth of endothelial cells.

### Hemin-binding proteins

The genome of *B. bacilliformis* encodes three hemin binding protein (*hbp*) genes [57] that are homologous to the Pap31 protein of *B. henselae* [58] (NCBI accession numbers ABA60112.1, KZN22406.1, KZM38396.1, EKS45023.1, ABM44681.1). So far, no functional data of Hbps exist although experiments suggest that these proteins react with patient sera (with unclear specificity). Pap31 of *B. bacilliformis* seems to be an immunodominant protein [57] and, therefore, it was proposed as a candidate for potential vaccine development strategies [59]. In line with this, owl monkeys (*Aotus nancymaae*) experienced a four-fold increase of anti-Pap31 (anti-Hbp) IgM levels after infection with *B. bacilliformis* [60].

### Invasion-associated locus proteins A and B

Invasion-associated locus proteins A and B (IalA, IalB; NCBI accession numbers P35640.1 and P35641.1) are important for the invasion of *B. bacilliformis* into erythrocytes. Heterologous expression of these proteins in *E. coli* resulted in a strong (up to 39-fold) increase of human erythrocyte invasion *in vitro* [61]. Homologous proteins have been found in other invasive bacteria (e.g. Ail of *Y. enterocolitica* mediating invasion into epithelial cells [62, 63]). The exact biological function of IalA, a (di)nucleoside polyphosphate hydrolase, is not clear [64]. The *ialB* gene encoding a membrane protein is highly conserved among other human-infecting *Bartonella* and an *ialB*-deficient mutant exhibits a decreased invasion in human erythrocytes [65]. The highest levels of *ialB* mRNA and IalB expression were found at 20 °C and acidic pH and the lowest levels were found at 37 °C and basic pH. These observations suggest that in chronic infections (verruga peruana), a further invasion of *B. bacilliformis* in circulating erythrocytes (which would result in hemolytic anemia) is avoided [66].

## Non-confirmed pathogenicity factors

### Deformin

**Fig. 7:**
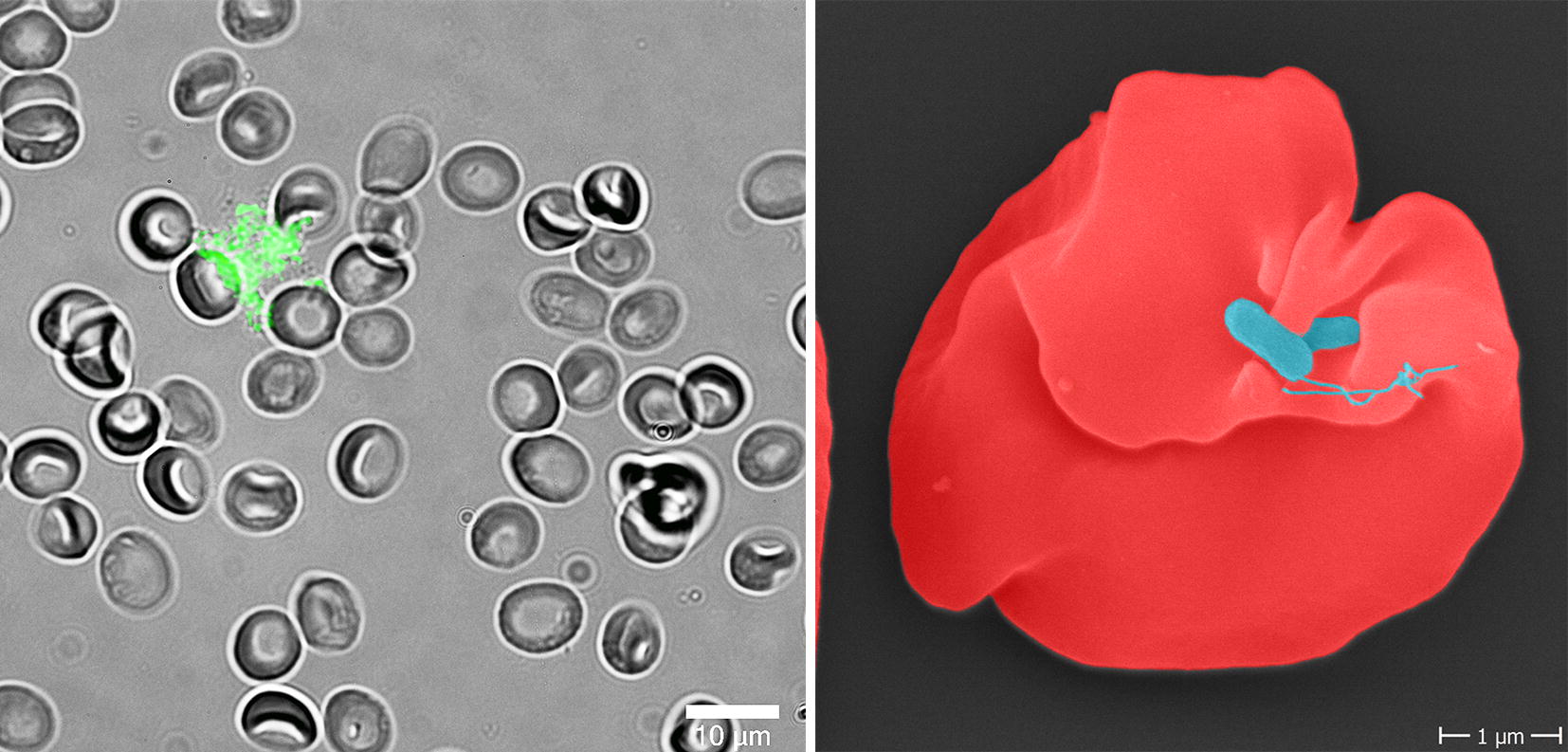
Human erythrocytes infected with *B. bacilliformis.* Left: Fluorescence microscopy of human erythrocytes infected with GFP-expressing *B. bacilliformis* ATCC 35686 (6 h). Note the deformation of the erythrocyte cell surface (Aepfelbacher and Kempf, 2018). *Scale-bar*: 10 µm. Right: Scanning electron microscopy of infected human erythrocytes (24 h). Note the deformation of the erythrocyte. *Scale-bar*: 1 µm (courtesy of C. Sittmann, Goethe University, Frankfurt am Main, Germany and K. Hipp, Max Planck-Institute for developmental Biology, Tuebingen, Germany)

An infection with *B. bacilliformis* induces morphological changes of erythrocytes that finally result in *Bartonella* invagination (Fig. 7). This deformation seems to be induced by extracellular molecules potentially secreted by *B. bacilliformis* (called “deformation factors” or “deformins” [47]). This effect was also detectable when erythrocytes were exposed to unknown compounds filtrated from *B. bacilliformis* culture supernatants. The nature of these compounds seems to be aminoacidic as heating of the supernatants prohibits this effect. To date, there is no consensus in the weight of the hypothetical molecule [67]. Moreover, in the recently published genomes, no clear hit for a “deformin” has been found.

### Hemolysins

The first deeper analysis of the hemolytic activity of *B. bacilliformis* revealed that for the hemolytic activity a proteinaceous compound might be responsible which increases red blood fragility but the author failed in an exact identification of the presumed compound [68]. Different accession numbers for hemolysin A and D are given (NCBI accession numbers KZN22078.1, KZM38023.1, EKS44973.1, KZN22169.1, KZN21496.1, KZM38155.1, KZM37455.1, ABM44735.1); however, these entries have not been supported by any functional data.

### Non-identified outer membrane proteins

Outer membrane proteins (OMPs) of *B. bacilliformis* were investigated for the ability to bind directly to actin. Six major proteins with molecular weights of 100, 92, 84, 46, 37 and 12 kDa, respectively, bind possibly to actin [69]. These experiments were limited by the fact that they were performed under SDS-denaturing conditions and no further functional assays have been published in course, neither were these proteins further identified. On the other hand,

*B. bacilliformis* was demonstrated to be able to bind human erythrocyte proteins such as spectrin, band 3 protein, and glycophorin A and B [70] which are components of the erythrocyte cytoskeleton.

## Cellular *B. bacilliformis* infection models

To date, no reliable small animal infection model exists for *B. bacilliformis*. Therefore, “cellular microbiology” seems to be the tool of choice to understand the underlying pathogenicity mechanisms occurring in *B. bacilliformis* infections. To study the biphasic Carrion’s disease, various *in vitro* infection models have been established employing erythrocytes and endothelial cells.

## Erythrocyte infection models

*Bartonella bacilliformis* infection experiments with human erythrocytes allow the analysis of bacterial adhesion and invasion in greater detail. For this, standard techniques were mainly employed [46, 71] as follows (or similar): after removing unbound bacteria by washing, erythrocyte-bound bacteria are visualized and quantified by Giemsa staining and light microscopy or *via* electron microscopy. By this, it was shown that *B. bacilliformis* leads to substantial and long-lasting deformations in erythrocyte membranes where bacteria are localized [46, 48] and this resulted in the hypothesis of a so-called “deformin” protein (see above). The entry of *B. bacilliformis* into erythrocytes has also been monitored by fluorescence microscopy and by transmission electron microscopy [46]. Moreover, invasion kinetics were determined using gentamicin-protection assays killing the extracellular bacteria prior the lysis of erythrocytes and subsequent cultivation of the intracellular (aminoglycoside-protected) bacteria [48]. Various studies revealed that non-motile or flagella-function-inhibited bacteria are drastically reduced in their association with erythrocytes. Furthermore, treatment with enzymes (affecting outer proteins) or incubation with respiratory chain inhibitors was also demonstrated to influence bacterial erythrocyte adherence [71].

## Endothelial cell infection models

*Bartonella bacilliformis* invades endothelial cells and induces cellular proliferation (similar to angiogenesis events) causing the formation of verruga peruana. To identify potential pathogeny factors, live bacteria, bacterial lysates or conditioned media were co-cultivated with human endothelial cells.

By using ^35^S-methionine-labeled bacteria, it has been shown that *B. bacilliformis* invades several cell types *in vitro* (e.g. human dermal fibroblasts, HEp-2 and HeLa-229 cells and HUVECs). From this it was hypothesized that the *in vivo* preference for endothelial cell infection might be based on the dissemination route (bloodstream) rather than on cell tropism [72]. Electron microscopy revealed that bacteria invade endothelial cells rapidly (1 h) forming large vacuolic inclusions after 12 hours of infection similar to Rocha-Lima inclusions [73]. *Bartonella bacilliformis* stimulates its entry into endothelial cells by activating Rho-family GTPases (Rho, Rac, Cdc42) leading to morphological changes of infected endothelial cells [74–76]. These small GTP-binding proteins are key regulators in the organization of the actin cytoskeleton and their activation results in the formation of filopodia and lamellopodia facilitating bacterial entry into host cells [76].

The addition of *B. bacilliformis* culture extracts stimulates HUVEC proliferation ~3-fold and this phenomenon was attributed to a heating-sensitive compound of around 12–14 kDa [77]. In addition, *B. bacilliformis* activates the release of the tissue plasminogen activator (t-PA) from endothelial cells *in vitro* and this process is known to be involved in angiogenic processes. These authors also demonstrated that infection with *B. bacilliformis* results in endothelial proliferation and that a direct contact between bacteria and host cells results in higher proliferation rates compared with settings where bacteria and host cells were physically separated [73]. The increase of endothelial proliferation (6- to 20-fold) was confirmed in a later study by exposing endothelial cells to *B. bacilliformis* culture supernatants and this phenomenon was dependent on a bacteria-derived proteinaceous mitogen [52].

Other experiments demonstrated that a *B. bacilliformis* infection results in a strong induction of angiopoietin-2 in endothelial cells [78]. These findings are in-line with the observations made by *in situ* hybridizations of clinical human verruga peruana specimens where high expression levels of angiopoietin-2 and vascular endothelial growth factor (VEGF) receptors were detected in the endothelium. As the major source of VEGF, the overlying epidermis of the verruga peruana was identified suggesting an angiogenic loop mechanism between infected endothelium and the overlying epidermis [78].

## Animal *B. bacilliformis*-infection models

Animal infection models are crucial to understanding bacterial pathogenicity mechanisms *in vivo*. Besides humans, only rhesus macaques are known to be susceptible to Carrion’s disease. In a study of Noguchi and Battistini from 1926, *Macacus rhesus* monkeys suffered from Oroya fever and verruga peruana illnesses after being infected with *B. bacilliformis* [79]. However, to date there is no reliable small animal *B. bacilliformis* infection model available. As a trade-off, particular laboratory parameters and the underlying immune response are determined by using blood and serum samples from infected patients. Not surprisingly, these samples are difficult to obtain and strongly limited by nature. Therefore, a suitable animal infection model is urgently needed.

The intravenous injection of *B. bacilliformis* in rhesus monkeys induced a prolonged irregular remittent fever. The pathogen was cultivatable from peripheral blood for a long period (58 days) [80] and was detected within erythrocytes, reproducing the precise appearances observed in human cases of Oroya fever. However, in all tested subjects the intensity of the anemia was less severe than in humans. The intradermal injection of *B. bacilliformis* resulted in nodular formations rich in new blood vessels where the bacteria were found within endothelial cells and could be re-isolated. Complete convalescence of the infected animals occurred after a period from two to five months [81]. Further experiments on rhesus monkeys showed that virulence of *B. bacilliformis* was enhanced by passaging the pathogen through susceptible animals. Here, a severe anemia with reduction of erythrocyte counts was observed but the number of invaded erythrocytes was still lower compared to Oroya fever in humans [80]. Furthermore, a high variety in the course of disease was observed: rhesus monkeys developed from mild (mild anemia, mild course of verruga peruana-like lesions) to severe (see above) symptoms after *B. bacilliformis* infections [80]. The variation of the course of infection suggested that the severity of symptoms of Carrion’s disease was primarily attributed to the virulence of the particular *B. bacilliformis* strain and secondarily depended on the (genetic) predisposition of monkeys [82]. The pathologic changes in the organs of monkeys suffering from a severe course of Carrion’s disease showed high similarity to those found in human organs of fatal cases. After the death of the animals, bacteria were re-isolated from the lymphatic system, spleen, bone marrow and liver [80]. Noguchi & Battistini undertook further attempts to identify animal species susceptible to *B. bacilliformis* infection (dogs, donkeys, guinea pigs, java, mice, rabbits, rats, ringtails, green monkeys, chimpanzees and orangutans) but only chimpanzees and orangutans showed clinical symptoms characteristic for Carrion’s disease [83, 84]. However, compared to rhesus monkeys, the severity of symptoms was much weaker and showed less resemblance to Carrion’s disease of humans [83]. Similar results were obtained ~80 years later by infecting owl monkeys. Here, these monkeys suffered also from a microscopically-detected intraerythrocytic bacteremia upon an intravenous *B. bacilliformis* infection; nevertheless (and for unclear reasons), detection of *B. bacilliformis via* cultures and PCRs remained negative [60]. To the best of our knowledge, today the *B. bacilliformis* monkey-infection model is no longer applied (most likely because of animal protection reasons and economic aspects).

There have been attempts to establish a rat infection model to determine the responsible mechanism of *B. bacilliformis* for inducing vascular proliferations [77]. Here, polyvinyl alcohol sponge discs were subcutaneously implanted into adult Sprague-Dawley rats and were injected with *B. bacilliformis* culture extracts three days after implantation. Sponges were analyzed microscopically after seven days and a ~2.5-fold increase in blood vessel formation was found. It needs to be mentioned that this rat model was established for the artificial application of *B. bacilliformis* extracts not reflecting the natural course of infection [77]. In another experimental setting, BALB/c mice were intraperitoneally, intradermally or subcutaneously inoculated with various amounts of viable *B. bacilliformis*, but histopathological lesions were not detected. Moreover, no bacteremia was detected for a period of 15 days after inoculation [85], reflecting that BALB/c mice are not an appropriate *B. bacilliformis* animal infection model. The lack of virulence of *B. bacilliformis* in murine infection models can be best explained by the absence of a Trw type 4 secretion system (Trw T4SS): it was shown that a distinct Trw locus of the respective animal-pathogenic *Bartonella* species is crucial for facilitating host-restricted adhesion to erythrocytes [86].

A potential alternative to mimic at least the bacteremia phase of a *B. bacilliformis* infection in humans (Oroya fever) and to overcome the species barrier in murine infection models is the use of so-called “humanized” mice. The engraftment of NOD-scid IL2rɤ^-/-^ mice with human hematopoietic stem cells results in *de novo* generation of human erythrocytes and such models have been used in analyzing e.g. the course of *Plasmodium falciparum* infections [87]. As *B. bacilliformis* is adapted to infect human erythrocytes, this promising model would probably enable to analyze some bacterial pathogenicity mechanisms. Nevertheless, in such humanized mice, endothelial cells (which represent the potential niche for *B. bacilliformis*) remain of murine origin and it is unknown how the murine-endothelial-cell origin affects the course of infection.

## Host immune response upon *B. bacilliformis* infections

Only little information exists about immunity in Carrion’s disease and immune response to *B. bacilliformis* infections. Reasons for this are the low availability of samples from the endemic areas, a hardly existing scientific attention to the disease and the lack of suitable animal infection models. There is moderate evidence that humoral and cellular immune responses are involved during Carrion’s disease. It is known that an infection with *B. bacilliformis* results in a lifelong humoral immunity which confers partial immunological protection [88] and this is in-line with earlier results showing that rhesus monkeys and chimpanzee which had recovered from an infection with *B. bacilliformis* showed complete immunity when repetitively infected [81].

Groundbreaking findings from 1929 are still valid today [89]: to study the effects of immune sera on the course of *B. bacilliformis* infections, rabbit immune sera and convalescent sera from infected rhesus monkeys were tested in infections of rhesus macaques. In most cases, convalescent sera delayed the formation of verruga peruana and inhibited a proliferative blood-stream infection with *B. bacilliformis* when simultaneously applied with the pathogen. The injection of convalescent sera after *B. bacilliformis* infections resulted in negative blood cultures but showed no effect on the formation of skin lesions.

In endemic regions, seropositivity (IgM, IgG) of humans can reach ~30–35%. Recent studies reported that the number of asymptomatic *B. bacilliformis* carriers is ~37% in post-outbreak areas and ~52% in endemic areas [51]. These asymptomatic individuals seem to represent the main reservoir of the pathogen. In an attempt to identify serum biomarkers to detect *B. bacilliformis* infections it was suggested to consider IgM as a marker of a recent infection and IgG as a marker of past exposure and immunity [88]. It was also shown that IgM levels correlate with low levels of eotaxin, IL-6 and VEGF and high levels of interleukin 10 (IL-10), reflecting an immunosuppression in the acute phase of Oroya fever [88]. IL-10 is a potent anti-inflammatory cytokine that plays a crucial role in limiting the host immune response to pathogens in order to prevent host damage. It was reported that some pathogens are able to utilize the immunosuppressive properties of IL-10 to limit the host immune response [90]. A decrease of the cellular mediated immune response and increased levels of IL-10 were also observed in two pregnant patients that suffered from a severe bartonellosis [91]. It is believed that *B. bacilliformis* induces a long lasting immunosuppression continuing after the acute phase (Oroya fever) and during the chronic phase of Carrion’s disease [88]. Due to this, levels of T_H_1-related and pro-inflammatory cytokines are reduced leading to persistent infections characterized by a low level-bacteremia [88]. Furthermore, the proangiogenic cytokines VEGF and eotaxin showed a positive correlation with IgG levels and a negative correlation with IgM levels in seropositive patients [88]. It has been demonstrated that *B. henselae* induces VEGF production *in vitro* and *in vivo* [92, 93]. It is hypothesized that with an enhanced IgG response, *B. bacilliformis* evades the immune system in endothelial cells to hide and replicate in this immunoprivileged niche [88].

## Conclusions

Carrion’s disease is an ancient disease. There is a worrisome lack of knowledge about vectors and possible reservoir hosts of *B. bacilliformis*. Insights into the dynamics of pathogen transmission by *Lutzomyia* species might help to gain prevention strategies. Clearly, a rigorous screening of the wildlife (animals and plants) would discard or confirm the existence of other *B. bacilliformis* reservoir hosts apart from human beings. Molecular mechanisms underlying host infections are also widely unknown. The use of appropriate *in vitro* and *in vivo* infection models in combination with molecular strategies using bacterial mutants (e.g. generated by random and targeted mutagenesis) and recombinant protein expression strategies (e.g. *via* heterologous expression libraries) could help to gain deeper insights into the infection biology of this difficult to handle pathogen and might represent a basis for the development of a potential vaccine.

## Abbreviations

DNA: deoxyribonucleic acid
GFP: green-fluorescent protein
HUVEC: human umbilical vein endothelial cells
IgG: immunoglobulin G
IgM: immunoglobulin M
IL-10: interleukin 10
MLST: multi-locus sequence typing
mRNA: messenger ribonucleic acid
NF-κB: nuclear factor κB
OMP: outer membrane protein
PCR: polymerase chain reaction
TAA: trimeric autotransporter adhesion
T_H_1: T helper 1
TLR5: Toll-like receptor 5
t-PA: tissue plasminogen activator
T4SS: type IV secretion system
VEGF: vascular endothelial growth factor
